# How many truck drivers have sleep disorders? Investigation of the effects of lifestyle and stress on insomnia among Japanese male truck drivers

**DOI:** 10.1093/joccuh/uiaf012

**Published:** 2025-02-17

**Authors:** Ryoya Aoki, Takashi Miyachi, Yuta Sugano, Choichiro Kanke, Teiichiro Yamazaki, Kazuo Mishima, Kyoko Nomura

**Affiliations:** Department of Medicine, Akita University Faculty of Medicine, Akita, Japan 1–1–1 Hondo, Akita, 010-8543, Japan, Department of Environmental Health Science and Public Health, Akita University Graduate School of Medicine, Akita, Japan 1–1–1 Hondo, Akita 010–8543, Japan; Department of Medicine, Akita University Faculty of Medicine, Akita, Japan 1–1–1 Hondo, Akita, 010-8543, Japan, Department of Environmental Health Science and Public Health, Akita University Graduate School of Medicine, Akita, Japan 1–1–1 Hondo, Akita 010–8543, Japan; Department of Medicine, Akita University Faculty of Medicine, Akita, Japan 1–1–1 Hondo, Akita, 010-8543, Japan, Department of Environmental Health Science and Public Health, Akita University Graduate School of Medicine, Akita, Japan 1–1–1 Hondo, Akita 010–8543, Japan; Department of Neurosurgery, Hadano Red Cross Hospital, Kanagawa, Japan 1–1 Tatsunodai, Hadano 257–0017, Japan; Department of Medicine, Akita University Faculty of Medicine, Akita, Japan 1–1–1 Hondo, Akita, 010-8543, Japan, Department of Environmental Health Science and Public Health, Akita University Graduate School of Medicine, Akita, Japan 1–1–1 Hondo, Akita 010–8543, Japan; Department of Neuropsychiatry, Akita University Graduate School of Medicine, Akita, Japan 1–1–1 Hondo, Akita 010–8543, Japan; Department of Medicine, Akita University Faculty of Medicine, Akita, Japan 1–1–1 Hondo, Akita, 010-8543, Japan, Department of Environmental Health Science and Public Health, Akita University Graduate School of Medicine, Akita, Japan 1–1–1 Hondo, Akita 010–8543, Japan

**Keywords:** caffeine, driving characteristics, insomnia, restless legs syndrome, sleep apnea syndrome, truck drivers

## Abstract

**Objectives:**

This study aimed to investigate how many male truck drivers have sleep disorders and what factors are most associated with chronic insomnia symptoms.

**Methods:**

A cross-sectional study of 505 truck drivers in Akita prefecture was conducted using a self-administered questionnaire and health checkup data. We defined insomnia based on the International Classification of Sleep Disorders, third edition, sleep apnea syndrome (SAS) with a simple 4-variable screening tool, and restless legs syndrome (RLS) with RLS/Willis-Ekbom disease diagnostic criteria. Investigated factors included sleep duration, driving characteristics, caffeine types (foods and beverage) and amounts, caffeine intake timing, State-Trait Anxiety Inventory (STAI), individual stress, and other covariates.

**Results:**

The prevalence of suspected SAS was 23.2% (*n* = 154), and that of RLS was 0.8% (*n* = 5). After excluding those, chronic insomnia symptoms were present in 36/505 drivers (7.1%). After adjusting for covariates, a logistic model demonstrated that drinking habits [odds ratio (OR), 6.21; 95% CI, 1.07-35.8], caffeine intake before sleep (OR, 2.65; 95% CI, 1.09-6.45), sleep duration on days off (OR, 1.44; 95% CI, 1.01-2.05), and STAI score (OR, 12.8; 95% CI, 2.53-64.2) were significantly associated with chronic insomnia symptoms. STAI was significantly positively correlated with individual stress, such as family worries (*r* = 0.22), relationships with non-partners (*r* = 0.28), and health (*r* = 0.23).

**Conclusions:**

Our study revealed that one-fourth of male truck drivers had sleep disorders that require further medical evaluation. For male truck drivers, lifestyle modification and stress relief may be key to address insomnia.

## Teaser

Key points

**What is already known on this topic:** Sleep problems are common among truck drivers, yet their prevalence and the comprehensive risk factors of sleep disorders are not well documented.

**What this study adds:** This study found that insomnia, sleep apnea syndrome, and restless legs syndrome are prevalent in this population. Caffeine intake before sleep and individual stress related to family, relationships, and health were associated with insomnia among truck drivers.

**How this study might affect research, practice, or policy:** Our study suggests that a screening opportunity should be provided for every single truck driver for road safety, and that lifestyle modification and stress relief may be key to addressing insomnia.

## 1. Introduction

As the domestic e-commerce market expands, further influenced by the COVID-19 pandemic, demand in the transportation industry is increasing. In 2023, monthly average working hours for Japanese truck drivers exceeded 200 hours, approximately 20% longer than the average of other occupations.^1^ The Japanese government revised the Labor Standards Act, limiting commercial drivers’ annual overtime to 960 hours from 2024. However, this regulation still represents 2.6 times the overtime allowed for general workers. Such occupational trends make drivers vulnerable to mental health problems, with over 50% admitting to feeling drowsy while driving.^2^ An interview survey of 60 truck drivers identified numerous stressors in their working conditions, including time constraints, social isolation, condescending treatment by clients, and driving hazards, like weather changes, traffic congestion, and road conditions.^3^ Mental health problems are thus prevalent among truck drivers, with 26.9% experiencing depression and 14.5% experiencing anxiety.^4^

This harsh working environment of truck drivers, and the resulting stress and anxiety, can impair sleep. Our previous study observed that 13.3% of Japanese truck drivers reported insomnia symptoms, significantly higher than the 3.2% in the general population.^5^ We further revealed that truck drivers with insomnia symptoms were more likely to have hypertension, diabetes mellitus, or dyslipidemia based on their annual health examinations or health insurance claims data.^6^^,^^7^ Drivers’ sleep condition affects both their well-being and public road safety, as drivers with insomnia carry twice the risk of traffic accidents.^8^ Due to their size and weight, truck collisions pose a 2 to 3 times higher fatality risk compared with passenger car accidents.^9^ In Japan, 391 fatalities were attributed to truck-related accidents in 2022.^10^ The increasing average age of truck drivers raises concerns regarding health-related incidents, such as sudden illness or falling asleep at the wheel. In 2022, the Japanese Ministry of Land, Infrastructure, Transport and Tourism reported 106 truck accidents caused by drivers’ health conditions.^11^

Truck drivers experience monotonous, prolonged driving and often rely on caffeine to maintain alertness, with 50% to 90% using it as a countermeasure against sleepiness.^12^^,^^13^ A cross-sectional questionnaire survey demonstrated a positive correlation between driving hours and caffeine consumption.^14^ Insufficient rest areas along highways and compensation systems like piece-rate pay further motivate drivers to extend driving hours while relying on caffeine intake.^2^^,^^15^^,^^16^ Several studies have investigated the relationship between caffeine consumption and driving performance, including its impact on traffic accident rates. An experimental driving study using a static vehicle on a virtual road showed that ingestion of 200 mg of caffeine significantly reduced driving incidents and subjective sleepiness.^17^ A case-control study also found that drivers consuming caffeine to stay alert were less likely to cause traffic accidents.^18^ Excessive caffeine intake can impair both the quantity and quality of nighttime sleep by prolonging sleep latency and decreasing slow-wave sleep.^19^ A study involving 96 female students observed that consuming 300 mg of caffeine 1 hour before bedtime increased nighttime worry and sleeplessness.^20^ These findings indicate that drivers’ use of caffeine to overcome drowsiness may ironically induce insomnia, causing daytime sleepiness. The timing of caffeine consumption is another critical factor in its impact on sleep disturbances, particularly given the irregular sleep schedule of truck drivers. However, few studies have investigated the relationship between caffeine intake timing and sleep quality among truck drivers.^21^ This study aimed to address this gap by investigating both the timing and quantity of caffeine intake through a comprehensive questionnaire. Additionally, although increased stress and worry can disrupt drivers’ sleep patterns, the specific sources of stress and worry among truck drivers remain unknown. Therefore, the purpose of this study was to identify the factors, including previously known conventional risk factors, most strongly associated with insomnia in Japanese truck drivers.

## 2. Materials and methods

### 2.1. Participants

In 2020, the Akita Trucking Association had 7200 trucks. Self-administered questionnaires and informed consent forms were distributed, and 2927 drivers, over 50% of truck drivers in Akita prefecture (*n* = 6150) per the 2020 Population Census (https://www.e-stat.go.jp/dbview?sid=0003464349), agreed and responded. Nevertheless, the majority of these were excluded because health checkup data were unavailable at the Japan Health Insurance Association, Akita branch (*n* = 1901). This occurred due to 2 reasons: some respondents might have been new drivers, and no data-sharing agreement existed between the checkup institutions and the Association. After excluding those whose data were not provided due to technical issues, such as insufficient workforce or lack of a digital system (*n* = 128), and other insurance subscribers (*n* = 9), we obtained health checkup and health insurance claims data for 889 individuals. In 2021, an additional questionnaire was distributed, with 683 individuals responding. Participants who were female (*n* = 9), of unknown sex (*n* = 6), or non–truck drivers (*n* = 5) were excluded, leaving 663 male truck drivers for analysis. The study design and data collection procedures were detailed in our previous report.^5^

### 2.2. Outcomes

Chronic insomnia symptoms were defined based on the diagnostic criteria of the International Classification of Sleep Disorders 3 (ICSD-3). Participants were considered to have chronic insomnia symptoms if they experienced at least 1 of the following: difficulty initiating sleep, difficulty maintaining sleep, or early morning awakenings at least 3 times a week for 3 months, accompanied by daytime dysfunction. Daytime episodes include fatigue, reduced attention, minor errors, depressed mood, irritability, daytime sleepiness, abusive behavior, decreased motivation, elevated risk of accidents, and sleep-related worry or anxiety. These were obtained in the 2021 questionnaire.

Daytime symptoms may be explained by other sleep disorders, such as sleep apnea syndrome (SAS) and restless legs syndrome (RLS), which often coexist with insomnia. To exclude these conditions, a simple 4-variable screening tool^22^ and RLS/Willis-Ekbom disease diagnostic criteria^23^ were employed. The SAS screening tool, with a sensitivity of 0.93 and a specificity of 0.66 at a cutoff point of 11, was deemed suitable for excluding suspected SAS cases.

### 2.3. Self-administered questionnaire in 2020

The 2020 questionnaires included the following items: smoking habits (never, past, current), driving distance (short-haul, middle-haul, long-haul), consecutive days away from home (not applicable, 1 day, 2 days or longer), daily driving hours (less than 8 hours, 8-9 hours, 10-11 hours, 12 hours or more), and drinking habits, categorized as nondrinker, normal (ethanol intake ≤483 g/wk), or heavy (ethanol intake >483 g/wk).

### 2.4. Health checkup data in 2020

We obtained each participant’s mandatory annual health checkup data, as required by the Industrial Safety and Health Act, from the Japan Health Insurance Association Akita Branch. The items collected were body mass index (BMI), systolic and diastolic blood pressure, fasting blood glucose, triglycerides, low-density lipoprotein (LDL) cholesterol, and medication histories for hypertension, diabetes, and dyslipidemia. BMI was classified into 2 categories (<25 kg/m^2^, ≥25 kg/m^2^). Hypertension was defined as systolic blood pressure ≥140 mmHg, diastolic blood pressure ≥90 mmHg, and/or use of antihypertensive medication. Dyslipidemia was defined by any of the following: triglyceride ≥150 mg/dL, high-density lipoprotein (HDL) cholesterol <40 mg/dL, LDL cholesterol ≥140 mg/dL, and/or use of antihyperlipidemic medications. Diabetes mellitus was defined as fasting blood glucose ≥126 mg/dL, and/or use of antidiabetic medications. Definitions of each lifestyle-related disease were described previously.^7^

### 2.5. Self-administered questionnaire in 2021

#### 2.5.1. Caffeine consumption

Participants were asked about their daily consumption of caffeine-containing beverages and foods, including green tea, oolong tea, black tea, coffee, energy drinks, menthol gum, caffeine drops, chocolates, and other products. For caffeine-containing beverages, consumption was assessed as the number of cups, cans, or bottles per day on a scale of 200 mL, 350 mL, and 500 mL. For caffeine-containing foods, intake was measured by the number of products (eg, pieces for candy or gum). The caffeine content in each product was obtained from the Japan Food Safety Commission.^24^ As the report did not include data for Japanese tea, milk tea, or café au lait, their caffeine content was estimated based on major manufacturers: 20 mg/100 mL for Japanese tea and milk tea, 40 mg/100 mL for café au lait. Considering the variations in caffeine content among energy drinks and chocolate products, participants were instructed to specify the names of the energy drinks or chocolates that they commonly consumed. When specific product names were indicated, their caffeine content was obtained from the manufacturer’s website. If details were unavailable, standardized caffeine contents of 36 mg/100 mL for energy drinks and 28 mg/100 g for chocolate were employed, based on information from major companies. Total daily caffeine consumption was calculated by summing the caffeine content from all sources. Participants were also asked if they consumed caffeine-containing beverages or foods within 4 hours before sleep, following guidelines of the Japanese Ministry of Health, Labour, and Welfare.

#### 2.5.2. State-Trait Anxiety Inventory

A validated Japanese version of the State-Trait Anxiety Inventory (STAI), the most widely used clinical anxiety rating scale, was used to assess anxiety.^25^ State anxiety, characterized as a temporary emotional reaction to stressful situations involving nervousness and anxious thoughts, was used to ascertain truck driver’s anxiety related to driving. Responses were evaluated using a 4-point Likert scale, from “almost never” (1) to “almost always” (4), with total scores categorized into quartiles.

#### 2.5.3.Stress while driving

To identify specific sources of stress while driving, brief interviews were conducted with members of the Akita Truck Association. The stressors identified included worries about being late to destinations, family matters, driving performance (ie, being overtaken), weather, traffic congestion, traffic accidents, falling asleep at the wheel, and other potential issues. Participants were then asked to indicate whether these concerns applied to them while driving. Responses were assessed using a 4-point Likert scale, ranging from “no” (1) to “very strong” (4).

#### 2.5.4. Stress in daily life

To assess stress in participants’ daily lives, they were also questioned about potential stressors, including financial situations (debt/mortgage), relationships with partners or others, health concerns, and other aspects of life. Responses were recorded on an 11-point Likert scale ranging from “not applicable” (0) to “applicable” (10).

#### 2.5.5. Covariates

Other covariates included age, length of service, and average daily sleep duration.

### 2.6. Statistical analyses

To assess the relationship between insomnia and the covariates, *t* tests or chi-square tests were applied based on the distribution of each variable. Logistic regression models were used to identify factors associated with insomnia. Odds ratios (ORs) were computed with 95% CIs. For multivariable logistic analyses, explanatory variables included factors significantly associated with chronic insomnia symptoms in the univariable analyses (ie, age, drinking habits, caffeine intake before sleep, driving distance, and STAI). Additionally, we included smoking habits, sleep duration on workdays and days off, and daily driving hours. The reasons for these are that smoking habits are often associated with a preference for caffeinated beverages and foods; a difference in sleep time between workdays and days off suggests sleep deprivation; and driving time may differ from the distance. We checked collinearity using the CORRB option in PROC logistic in SAS, which refers to a situation where 2 or more independent variables are highly correlated with each other, making it difficult to accurately estimate the individual effect of each variable on the dependent variable. We determined high collinearity when the correlation coefficient exceeded 0.8. Cronbach alpha and correlation coefficients with the STAI were calculated for stress while driving and stress in daily life, respectively.

All statistical analyses were performed using SAS 9.4. All the tests were 2-sided, and statistical significance was set at *P* < .05.

### 2.7. Ethical considerations

This study was approved by the Ethics Committee of the Akita University Graduate School of Medicine (approval number: 2456) and conducted in accordance with the Declaration of Helsinki. All participants provided informed consent. This study was supported by the Japan Organization of Occupational Health and Safety, the Akita Occupational Health Support Center, and the Japan Health Insurance Association, Akita branch.

## 3. Results

### 3.1.B aseline characteristics of truck drivers according to chronic insomnia symptoms

Of 663 male truck drivers, 154 (23.2%) drivers screened positive for SAS and 5 (0.8%) drivers screened positive for RLS. After excluding these cases, 505 truck drivers yielded a age of 53.6± 7.8 (mean±SD) years, and length of service of 23.7± 10.3 years. For smoking habits, 56.9% were current smokers, whereas nonsmokers made up only 9.6%. Heavy drinkers comprised 17.2%, and never-drinkers 21.3%. Sleep duration averaged 6.8 hours on workdays and 7.2 hours on days off, suggesting sleep deprivation. The STAI score was 44.4± 6.4 (mean±SD) points. Chronic insomnia symptoms were observed in 36 truck drivers (7.1%).

**Figure 1:**
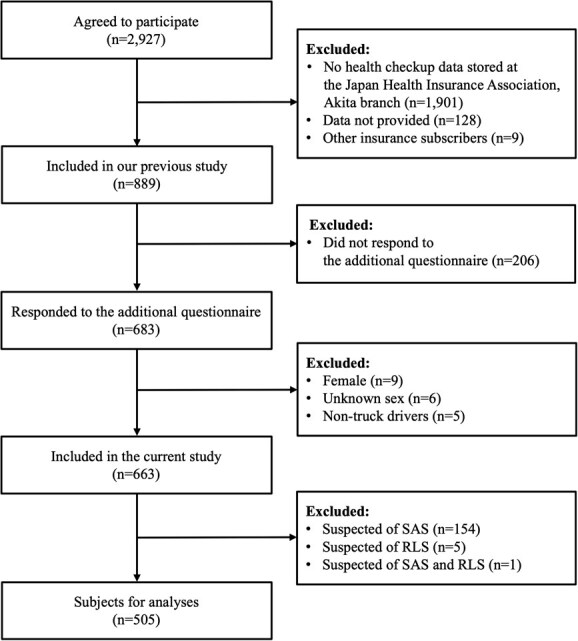
Flow diagram of participants.

Truck drivers with chronic insomnia symptoms were more likely to be heavy alcohol drinkers (*P* = .026), consume caffeine before sleep (*P* = .039), be middle-haul drivers (*P* = .035), and have higher STAI scores (*P* < .001; Table 1). The study flow diagram is shown in Figure 1.

**Table 1:** Baseline characteristics of truck drivers according to chronic insomnia symptoms.

		**Total**		**Chronic insomnia symptoms**				
		**(*n* = 505)**		**Present**		**Absent**		***P* value** ^**a**^
				**(*n* = 36; 7.1%)**		**(*n* = 469; 92.9%)**		
**Age,** ^**b**^ **y**		53.6 ± 7.8		51.3 ± 8.4		53.8 ± 7.7		.069
**Length of service,** ^**b**^ **y**		23.7 ± 10.3		23.7 ± 11.3		23.8 ± 10.2		.975
**Drinking habits,** ^**b**^ **g/wk**		229 ± 203		322 ± 275		222 ± 195		
	**Nondrinker**	105	(21.3)	3	(8.6)	102	(22.2)	.026
	**Normal (ethanol consumption ≤483)**	304	(61.5)	21	(60.0)	283	(61.7)	
	**Heavy (ethanol consumption >483)**	85	(17.2)	11	(31.4)	74	(16.1)	
**Smoking habits**								
	**Never**	48	(9.6)	2	(5.6)	46	(9.9)	.308
	**Past**	168	(33.5)	16	(44.4)	152	(32.7)	
	**Current**	285	(56.9)	18	(50.0)	267	(57.4)	
**Caffeine intake,** ^**b**^ **mg/d**		303 ± 205		292 ± 217		304 ± 204		
	**Normal (≤400)**	405	(80.2)	29	(80.6)	376	(80.2)	.956
	**Heavy (>400)**	100	(19.8)	7	(19.4)	93	(19.8)	
**Caffeine intake before sleep**								
	**(+)**	211	(44.8)	21	(61.8)	190	(43.5)	.039
	**(−)**	260	(55.2)	13	(38.2)	247	(56.5)	
**Body mass index,** ^**b**^ **kg/m**^**2**^		23.7 ± 2.8		23.5 ± 2.5		23.7 ± 2.8		
	**<25**	365	(72.3)	26	(72.2)	339	(72.3)	.994
	**≥25**	140	(27.7)	10	(27.8)	130	(27.7)	
**Sleep duration on workdays,** ^**b**^ **h**		6.7 ± 1.1		6.5 ± 1.4		6.7 ± 1.1		.283
**Sleep duration on days off,** ^**b**^ **h**		7.6 ± 1.2		7.8 ± 1.4		7.5 ± 1.1		.227
**Daily driving hours**								
	**<8**	287	(57.8)	17	(48.6)	270	(58.4)	.254
	**≥8**	210	(42.3)	18	(51.4)	192	(41.6)	
**Driving distance**								
	**Short-haul**	300	(59.4)	16	(44.4)	284	(60.6)	.035
	**Middle-haul**	100	(19.8)	13	(36.1)	87	(18.6)	
	**Long-haul**	105	(20.8)	7	(19.4)	98	(20.9)	
**Consecutive days away from home**								
	**Not applicable**	342	(67.7)	22	(61.1)	320	(68.2)	.636
	**1 day**	53	(10.5)	4	(11.1)	49	(10.5)	
	**2 days or longer**	110	(21.8)	10	(27.8)	100	(21.3)	
**State-Trait Anxiety Inventory (STAI) Score** ^**b**^		44.4 ± 6.4		50.1 ± 8.1		44.0 ± 6.1		<.001
	**Quartiles:**							
	**≤39**	98	(20.9)	2	(5.9)	96	(22.0)	.001
	**40 to ≤44**	145	(30.9)	6	(17.7)	139	(31.9)	
	**45 to ≤50**	161	(34.3)	12	(35.3)	149	(34.2)	
	**≥51**	66	(14.0)	14	(41.2)	52	(11.9)	
**Lifestyle-related diseases**								
	**Hypertension** ^**c**^	151	(29.9)	9	(25.0)	142	(30.3)	.505
	**Dyslipidemia** ^**d**^	303	(60.0)	19	(52.8)	284	(60.6)	.359
	**Diabetes** ^**e**^	33	(6.5)	2	(5.6)	31	(6.6)	.805

a *t* test for continuous variables or chi-square test for categorical variables.

b Values are means ± SD for continuous variables, or *n* (%) for categorical variables.

c Systolic blood pressure ≥140 mmHg, diastolic blood pressure ≥90 mmHg, and/or antihypertensive drug use.

d Triglyceride level ≥150 mg/dL, high-density lipoprotein cholesterol (HDL-C) <40 mg/dL, low-density lipoprotein cholesterol (LDL-C) level ≥140 mg/dL, and/or hypolipidemic agent use.

e Fasting blood glucose ≥126 mg/dL, and/or antidiabetic drug use.

### 3.2. Details of stress while driving and stress in daily life

Table 2 lists the correlation coefficients between the STAI and individual items of stress while driving and stress in daily life. Internal consistency was demonstrated by Cronbach alpha values of .763 for stress while driving and .865 for stress in daily life. Among stress while driving, family matters showed a weak correlation with STAI (*r* = 0.22, *P* < .001). In stress related to daily life, weak positive correlations with STAI were observed for relationships (*r* = 0.28, *P* < .001) and health concerns (*r* = 0.23, *P*< .001).

**Table 2:** Details and correlation analyses of stress while driving and stress in daily life.

		**Median (25th-75th percentile)**	**Correlation coefficient with STAI** ^**b**^	
**Stress while driving (α = .763** ^**a**^**)**				
	**Delay**	2 (1–2)	0.18	**
	**Family matters**	2 (1–2)	0.22	**
	**Being overtaken**	1 (1–2)	0.15	**
	**Weather and traffic jams**	2 (1–2)	0.18	**
	**Accidents**	2 (2–3)	0.17	**
	**Dozing**	1 (1–1)	0.18	**
	**Others**	1 (1–1)	0.36	
**Stress in daily life (α = .865** ^**a**^**)**				
	**Financial**	1 (0–5)	0.17	**
	**Partner**	0 (0–3)	0.10	
	**Relationships**	2 (0–5)	0.28	**
	**Health**	3 (0–5)	0.23	**
	**Others**	2 (0–5)	0.24	**

Abbreviation: STAI, State-Trait Anxiety Inventory.

a Values of Cronbach alpha within the items for each stress category.

b *P<.05, **P<.01.

### 3.3. Logistic regression analyses of chronic insomnia symptoms

In univariable logistic regression analyses, the *P* values for the important factors included in multivariable models were: age (*P* = .070), drinking habits (*P* = .036), smoking habits (*P* = .317), caffeine intake before sleep (*P* = .043), sleep duration on workdays (*P* = .185), sleep duration on days off (*P* = .227), daily driving hours (*P* = .257), driving distance (*P* = .042), and STAI score (*P* < .001).

Because of the high correlation between the STAI and stress while driving and stress in daily life, only the STAI was included as an explanatory variable.

The results of the multivariable logistic regression analysis for chronic insomnia symptoms are presented in Table 3. After including all predictive factors identified in univariable analyses, no high collinearity problems were observed in the model. Statistically significant factors associated with chronic insomnia symptoms were drinking habits, caffeine intake before sleep, and STAI score. The adjusted OR for heavy drinking was 6.21 (95% CI, 1.07-35.8); the adjusted OR for caffeine intake before sleep was 2.65 (95% CI, 1.09-6.45); the adjusted OR for sleep duration on days off was 1.44 (95% CI, 1.01-2.05); and the adjusted OR for the highest quartiles of STAI was 12.8 (95% CI, 2.53-64.2) compared with the lowest quartile.

**Table 3:** Logistic regression analyses of chronic insomnia symptoms.

		**Chronic insomnia symptoms**		
		**Crude OR**	***P* value**	**Adjusted OR (95% CI)**
**Age**		0.96	.070	0.95 (0.89–1.01)
**Length of service**		1.00	.974	
**Drinking habits**			.036	
	**Nondrinker**	ref		ref
	**Normal (ethanol consumption ≤483 g/wk)**	2.52		4.85 (0.99–23.7)
	**Heavy (ethanol consumption >483 g/wk)**	5.05		6.21 (1.07–35.8)
**Smoking habits**			.317	
	**Never**	ref		ref
	**Past**	2.42		1.46 (0.27–7.74)
	**Current**	1.55		0.51 (0.09–2.68)
**Caffeine intake**			.956	
	**Normal (≤400 mg/d)**	ref		
	**Heavy (>400 mg/d)**	0.98		
**Caffeine intake before sleep**			.043	
	**(−)**	ref		ref
	**(+)**	2.10		2.65 (1.09–6.45)
**Body mass index**			.994	
	**<25 kg/m** ^**2**^	ref		
	**≥25 kg/m** ^**2**^	1.00		
**Sleep duration on workdays**		0.81	.185	0.84 (0.57–1.25)
**Sleep duration on days off**		1.19	.227	1.44 (1.01–2.05)
**Daily driving hours**			.257	
	**<8 h**	ref		ref
	**≥8 h**	1.49		1.40 (0.53–3.64)
**Driving distance**			.042	
	**Short-haul**	ref		ref
	**Middle-haul**	2.65		1.67 (0.61–4.58)
	**Long-haul**	1.27		0.78 (0.23–2.60)
**Consecutive days away from home**			.639	
	**Not applicable**	ref		
	**1 day**	1.19		
	**2 days or longer**	1.46		
**State-Trait Anxiety Inventory (STAI)**			<.001	
	**≤39**	ref		ref
	**40 to ≤44**	2.07		1.70 (0.29–9.93)
	**45 to ≤50**	3.87		3.84 (0.77–18.9)
	**≥51**	12.92		12.8 (2.53–64.2)
**Lifestyle-related diseases**				
	**Hypertension** ^**a**^	0.77	.506	
	**Dyslipidemia** ^**b**^	0.73	.360	
	**Diabetes** ^**c**^	0.83	.806	

Abbreviations: OR, odds ratio; ref, reference.

a Systolic blood pressure ≥140 mmHg, diastolic blood pressure ≥90 mmHg, and/or antihypertensive drug use.

b Triglyceride level ≥150 mg/dL, high-density lipoprotein cholesterol (HDL-C) <40 mg/dL, low-density lipoprotein cholesterol (LDL-C) level ≥140 mg/dL, and/or hypolipidemic agents use.

c Fasting blood glucose ≥126 mg/dL, and/or antidiabetic drug use.

## 4. Discussion

This study investigated factors associated with chronic insomnia symptoms and specific stress experienced by Japanese male truck drivers. Chronic insomnia symptoms were observed in 36 drivers (7.1%) and were significantly associated with drinking habits, caffeine intake before sleep, sleep duration on days off, and the STAI score, whereas total daily caffeine intake showed no significant association. Additionally, stressors for truck drivers included family matters and various driving conditions during work, as well as financial concerns, relationships beyond their partners, and health problems in daily life. We discuss these findings, with reference to previous studies.

Our study found a 7.1% prevalence of chronic insomnia symptoms among participants, which is lower than previously reported rates. The prevalence of insomnia based on the Diagnostic and Statistical Manual of Mental Disorders, 4th edition was 7%.^26^ A study in France using the ICSD-3 found a 9.1% prevalence of chronic insomnia in men.^27^ Although few studies have examined chronic insomnia among truck drivers, a Korean study employing the Insomnia Severity Index (ISI), reported a 15.2% prevalence of insomnia among commercial motor vehicle drivers.^28^ However, the ISI does not evaluate chronic insomnia symptoms. In summary, the relatively low prevalence in our study might be explained by the exclusion of suspected SAS through the screening test.

Truck drivers with suspected SAS and RLS accounted for 23.2% and 0.8%, respectively. Among them, 18 drivers (2.7%) satisfied the criteria for both suspected SAS and chronic insomnia symptoms, whereas 2 drivers (0.3%) satisfied the criteria for both suspected RLS and chronic insomnia symptoms. Prevalence rates of these conditions can vary among studies owing to differences in diagnostic criteria. For instance, a study using the apnea-hypopnea index (AHI) among the general Japanese population showed that one-third of participants had SAS (AHI ≥ 5).^29^ Another study of Australian professional drivers using the respiratory disturbance index and Epworth sleepiness scale (ESS) reported an SAS prevalence of 15.8%.^30^ A previous study reported a 1.8% prevalence of RLS using the International Restless Legs Syndrome Study Group consensus criteria.^31^ Our findings highlight the high prevalence of sleep disorders among truck drivers, emphasizing the need for further screening for SAS and other sleep disorders in this population. Considering that both sleep disorders and insomnia are associated with an increased risk of traffic accidents, these results highlight the urgency of interventions to identify undiagnosed sleep disorders among truck drivers to improve public road safety.

We categorized caffeine consumption into 2 groups: consumption of 400 mg or more per day and less. A study suggested that caffeine intake exceeding 400 mg/d may cause chronic physiological arousal, potentially contributing to insomnia symptoms.^32^ The European Food Safety Authority also stated that regular caffeine intake of up to 400 mg/d is safe for adults.^33^ In our study, 19.8% of drivers consumed 400 mg or more of caffeine daily, exceeding the 15.4% reported for the general Japanese population.^34^ This discrepancy indicates that a considerable proportion of drivers rely on caffeine to maintain alertness while driving. However, no association was observed between total caffeine intake and chronic insomnia symptoms in our study. This result is not entirely consistent with previous studies. For instance, Filtness et al^35^ reported that truck drivers consuming 380 mg or more caffeine daily had shorter sleep durations and increased subjective sleepiness, measured by ESS, compared with those consuming only 1 cup of caffeine-containing beverages per day. A recent meta-analysis of young adults also demonstrated a robust association between increased caffeine consumption and sleep disturbances.^36^ Most studies in the meta-analysis assessed sleep quality using the Pittsburgh sleep quality index (PSQI).^36^ As PSQI is a screening tool for general sleep disorders rather than a diagnostic or severity assessment tool for insomnia, the actual effects of caffeine consumption on insomnia development remain uncertain. Furthermore, to our knowledge, very few studies have investigated the effects of caffeine consumption on insomnia in shift workers, including truck drivers. This study is the first to explore the association between caffeine intake and chronic insomnia symptoms in truck drivers using actual diagnostic criteria. Although no significant association was found, our findings underscore the need for further research to clarify the role of caffeine consumption in the occurrence of insomnia among truck drivers.

Truck drivers who consumed caffeine before sleeping were more likely to experience chronic insomnia symptoms, consistent with previous studies showing that the timing of caffeine intake affects sleep quality.^37^ Caffeine promotes alertness and has a half-life of 4-6 hours, with blood levels peaking approximately 30-45 minutes after ingestion.^38^ Referring to this characteristic, Japanese Ministry of Health, Labour, and Welfare advised drivers to avoid consuming caffeine within 4 hours of bedtime. However, a real-world study investigating the effect of caffeine consumption on sleep duration in 97 truck drivers reported contradictory results. The study showed that caffeine consumption within 5 hours before sleep did not significantly impact sleep duration.^21^ This study used objective data, employing actigraphy to measure sleep duration and recording the quantity and timing of caffeine consumption whenever drivers consumed caffeine-containing beverages or foods. Despite the objective nature of these data, the authors did not assess subjective sleep symptoms, which is a key criterion for diagnosing insomnia under the ICSD-3 criteria.

In our study, sleep duration on days off, rather than on workdays, was associated with an increased risk of chronic insomnia symptoms. The longer sleep duration on days off compared with workdays may reflect sleep deprivation due to insufficient sleep on workdays. This pattern may impair sleep quality, cognitive and motor functions while driving, reduce overall driving performance, and increase the risk of accidents.

Although an association between insomnia and anxiety among truck drivers has been previously reported,^5^ this study identified specific stressors linked to anxiety. Family matters and various driving conditions were significant stress factors while driving, correlating with higher STAI scores. These findings align with previous studies indicating that truck drivers face various occupational stressors.^3^ However, our study specifically highlights the link between the STAI score and family problems while driving. Notably, whereas family matters were significantly associated with the STAI score during driving, no such association was observed in the category of stress experienced in daily life. This suggests that truck drivers experience elevated anxiety related to family issues, particularly while driving. Previous studies have also revealed the strain of domestic discord owing to drivers’ prolonged absences from home, often owing to long-haul routes.^39^ Additionally, research suggests that positive relationships with friends and family can enhance sleep quality and promote safe driving decisions among truck drivers, whereas pressure from dispatchers to extend driving hours and fears of punitive actions negatively impact these outcomes.^40^ These findings demonstrate the intricate interplay of stressors contributing to insomnia in truck drivers, underscoring the need to consider these factors when addressing sleep issues in this profession.

The strength of this study included the use of an updated questionnaire to better assess the prevalence of chronic insomnia symptoms, with a particular focus on caffeine intake and stress. First, unlike our previous research, which did not clearly define insomnia symptoms, this study used an improved questionnaire that ruled out other sleep disorders, such as SAS and RLS, through valid screening and diagnostic tools. This refinement enabled a more accurate evaluation of the prevalence of chronic insomnia symptoms and other sleep disorders among truck drivers. Second, we accurately assessed the quantity of caffeine consumption in the current study. The previous questionnaire asked participants, “How many cups of each caffeinated beverage do you drink in a day?” However, we found that drivers consume caffeine more frequently in cans and bottles rather than cups, a factor incorporated into the current study. We also included the timing of caffeine intake, addressing the previously observed lack of significant effects observed for total weekly caffeine intake. Third, although our previous study found an association between STAI score and an increased risk of insomnia symptoms, the causes of anxiety and stress affecting drivers remained unclear. After consulting experts from the Track Association and reviewing relevant literature, we identified several psychological factors that could disturb truck drivers while on the road. These factors were incorporated into the current study’s questionnaire alongside the STAI score. Thus, these updates, combined with the improved methodology of this study, offer valuable contributions to the occupational health of Japanese truck drivers.

Nevertheless, this study has several limitations that must be addressed. First, its cross-sectional design prevents the establishment of causal relationships. Second, labor characteristics (ie, driving distance, consecutive days away from home, and daily driving hours) were obtained in 2020, whereas insomnia symptoms were measured in 2021. A 1-year time difference might influence the results. In fact, during 2020-2021, the COVID-19 pandemic led to increased logistics demands, possibly requiring drivers who typically transported goods short distances (eg, within the prefecture) to undertake long-distance travel. Additionally, weekly schedules may have varied under similar circumstances, potentially causing differential misclassification. Thus, labor characteristics may not have shown a significant association with insomnia symptoms. Third, the potential for misclassification or inaccuracies in several variables, particularly caffeine intake, should be considered due to reliance on a self-administered questionnaire. Fourth, the prevalence of insomnia symptoms in this study may have been underestimated owing to socially desirable and non-responder biases. Some drivers may have hesitated to report sleep issues, fearing that disclosure could negatively impact their employment. Similarly, participants might have underreported stress levels during driving and daily life to present themselves more favorably to employers. Fifth, our results could be confounded by mental health conditions such as depression, which can disrupt sleep, increase caffeine intake, and exacerbate anxiety. However, since the participants were currently employed, individuals with clinically significant mental health conditions were likely excluded. Nevertheless, future studies should consider assessing depression levels, as insomnia, anxiety, and stress could be attributed to underlying mental illness. Sixth, subjects identified as having chronic insomnia symptoms in our study might not fully meet the clinical diagnostic criteria for chronic insomnia, as a clinical diagnosis requires comprehensive history-taking and the exclusion of other sleep disorders. The questionnaire used to screen for suspected SAS and RLS might not be sufficient to rule out these conditions definitively. To address this limitation, we used the term “chronic insomnia symptoms” instead of “chronic insomnia.” Lastly, although we investigated labor characteristics, such as driving hours, driving distance, and consecutive days away from home, which were not significant, some critics may argue that other labor characteristics, such as truck size, type of product delivered, frequency of shifts worked, compensation methods, and highway use, should also have been included. However, incorporating all these factors into the questionnaire may reduce the response rate in a filed study, and could cause collinearity issues in multivariable regression analyses. Hence, we prioritized daily driving hours, driving distance categories, and consecutive days away from home over a broader range of work characteristics. Further research may address these additional factors through alternative approaches, such as qualitative analyses.

### 4.1. Conclusion

This study revealed that one-fourth of male truck drivers were suspected of having sleep disorders requiring further medical evaluation. Chronic insomnia symptoms among Japanese male truck drivers were associated with drinking habits, caffeine intake before sleep, and anxiety. Several stressors affecting truck drivers while driving, including family matters and various working conditions, were identified, although stress levels were not critically high. Although labor characteristics were not directly linked to insomnia symptoms, the 1-year time lag between the measurement of insomnia symptoms and labor characteristics may require further research on their potential impact. These results suggest the importance of lifestyle modifications and stress-focused interventions to reduce insomnia symptoms in this occupational cohort, ultimately enhancing road safety.

## Data Availability

The research data are available upon reasonable request to the corresponding author.
